# Gelenkersatz bei isolierter patellofemoraler Arthrose: Eine kritische Einordnung der Optionen

**DOI:** 10.1007/s00132-025-04654-3

**Published:** 2025-05-02

**Authors:** Alec Voordeckers, George Avram, Natalie Mengis, Laszlo Toth, Matthias Koch, Michael T. Hirschmann, Elias Ammann

**Affiliations:** 1 Department of Orthopedic Surgery and Traumatology, Kantonsspital Baselland, 4101 Bruderholz, Schweiz; 2 Department of Clinical Research, Research Group Michael T. Hirschmann, Regenerative Medicine & Biomechanics, University of Basel, 4001 Basel, Schweiz; 3 Zentrum Bewegungsapparat, Klinik für Orthopädie und Traumatologie des Bewegungsapparates, Team Knie und Sportorthopädie Kantonsspital Baselland, 4101 Bruderholz, Schweiz

**Keywords:** Kniegelenk, Knieteilprothese, Patella, Patellofemoralgelenk, Knietotalprothese, Knee joint, Partial knee replacement, Patella, Patellofemoral joint, Total knee replacement

## Abstract

**Hintergrund:**

Die isolierte patellofemorale Arthrose ist eine degenerative Erkrankung des Kniegelenks, die zu Schmerzen und teils immobilisierenden Funktionseinschränkungen führen kann. Ist die konservative Therapie ausgeschöpft, kann ein Gelenkersatz die Lebensqualität und Mobilität der betroffenen Patienten relevant verbessern. In solchen Fällen gilt es, spezifisch die am besten geeignete Knieprothese für den Patienten zu wählen. Dieser Artikel beleuchtet die Entscheidungsfindung zwischen patellofemoraler Prothese, Knietotalprothese und bikondylärem Ersatz (ohne Rückflächenersatz der Kniescheibe).

**Therapieoptionen:**

Die patellofemorale Prothese kommt vor allem bei jüngeren Patienten zum Einsatz und kann bei korrekter Implantation zu guten Resultaten und Hinauszögern eines totalprothetischen Ersatzes führen. Bei älteren Patienten mit isolierter patellofemoraler Arthrose wird meist das gesamte Kniegelenk ersetzt und eine Knietotalprothese gewählt, da diese zuverlässiger zu guten Ergebnissen führt und mit einer geringeren Revisionsrate verbunden ist. In ausgewählten Fällen muss insbesondere bei stark ausgedünnter Patella auf einen Retropatellarersatz verzichtet werden.

## Abstract (en)

**Background:**

Isolated patellofemoral osteoarthritis is a degenerative disease of the knee joint that may cause disabling pain and functional limitations. When conservative therapy has failed, arthroplasty can significantly improve the quality of life and mobility of affected patients. In such cases, it is important to select the most suitable implant for the patient on an individual basis. This article discusses the decision-making process between patellofemoral arthroplasty, total knee replacement, and bicondylar replacement without patellar resurfacing.

**Therapeutic options:**

Patellofemoral arthroplasty is mainly used in younger patients and, if implanted correctly, can lead to good results and a delay in the need for total knee replacement. In older patients with isolated patellofemoral osteoarthritis, total knee replacement is usually the preferred option due to its predictable good outcomes and a lower revision rate. In selected cases, particularly with severely thinned patella, retropatellar resurfacing should not be done.

Eine patellofemorale Arthrose kann zu Schmerzen und Funktionseinschränkungen führen. In vielen Fällen verschafft eine konservative Therapie Linderung. Sind hier aber die Möglichkeiten ausgeschöpft, kann ein Gelenkersatz die Lebensqualität verbessern. Dieser Artikel soll die Erfolgsaussichten der verschiedenen endoprothetischen Optionen zur Behandlung der isolierten patellofemoralen Arthrose beleuchten und bei der Entscheidungsfindung zum richtigen Gelenkersatz helfen

## Einleitung

Die isolierte patellofemorale Arthrose (PFA) ist eine degenerative Erkrankung des Patellofemoralgelenkes mit progressivem Knorpel-Knochen-Schaden der Patella und des Femurs, die zu Veränderungen der Biomechanik, Schmerzen und in der Folge Funktionseinschränkungen führt [1]. Es handelt sich hier um einen spezifischen Subtyp der Kniearthrose, der in X‑Beinen häufiger vorkommt als in geraden und O‑Beinen [2].

Die Prävalenz der PFA variiert von Population zu Population und wird klinisch oft unterschätzt. Radiologische Studien in bevölkerungsbasierten Kohorten beschreiben eine Prävalenz der PFA um 25 %, ohne nennenswerte Unterschiede zwischen Frauen und Männern. Bei Patienten mit Knieschmerzen liegt die Prävalenz einer PFA bei 39 %, wobei Frauen deutlich häufiger betroffen sind als Männer. Dies wird durch eine vermehrte ligamentäre Laxität und ein patellofemorales Maltracking beim weiblichen Geschlecht und der häufiger vorhandenen X‑Beinstellung bei Frauen erklärt [3].

Abgesehen vom Geschlecht stellen ein Patellamaltracking, rezidivierende Kniescheibenluxationen und generell eine Patellainstabilität Hauptrisikofaktoren der isolierten Arthroseentwicklung im patellofemoralen Gelenk dar. Die Biomechanik des patellofemoralen Gelenks ist komplex und viele anatomische Faktoren beeinflussen den Lauf der Kniescheibe und die patellofemorale Knorpelbelastung. Insbesondere gilt es auch, die tibiofemorale Stabilität zu berücksichtigen, die einen bedeutenden Einfluss auf die Belastung des Streckapparats und somit auch das patellofemorale Gelenk hat [4].

Bei der Therapie einer patellofemoralen Arthrose gilt es zwischen konservativen, gelenkerhaltenden und gelenkersetzenden Verfahren zu unterscheiden. Nichtchirurgische Behandlungsmöglichkeiten konzentrieren sich auf das Symptommanagement mit Physiotherapie, Taping, Orthesen und Aktivitätsanpassung. Begleitend können eine analgetische Therapie und intraartikuläre Injektionen mit Hyaluronsäure, PRP oder Kortison den Symptomverlauf positiv beeinflussen [5].

Gelenkerhaltende chirurgische Verfahren (z. B. Beinachsenkorrekturen, Versetzung der Tuberositas tibiae sowie Weichteilstabilisationen) werden angewendet, um das patellofemorale Tracking zu optimieren und somit die Druckbelastung im patellofemoralen Gelenk zu optimieren. Weitere operative Optionen fokussieren auf die Knorpelregeneration und versuchen, den Gelenkersatz hinauszuzögern oder zu vermeiden [6].

**Abb. 1:**
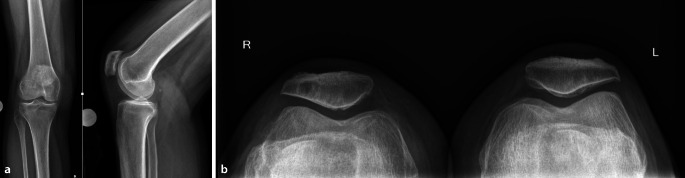
Konventionell-radiologische Bilder (**a** a. p., **b** lateral, Patella tangential) einer Patientin mit isolierter patellofemoraler Arthrose, jedoch konventionell-radiologisch nur leichten Arthrosezeichen (MRT in Abb. 2)

Ist die Arthrose allerdings stark fortgeschritten und sind nichtoperative Maßnahmen ausgeschöpft, kommen gelenkersetzende Operationen zum Einsatz. Es wird zwischen einer isolierten patellofemoralen Prothese (Kniescheibenrückfläche und Trochlea), einem bikondylären (Femur- und Tibia) und einem trikompartimentellen Ersatz (Femur‑, Tibia-, und retropatelläre Komponente) unterschieden [7]. Dieser Artikel soll die Erfolgsaussichten der verschiedenen endoprothetischen Optionen zur Behandlung der isolierten patellofemoralen Arthrose beleuchten und bei der Entscheidungsfindung zum richtigen Gelenkersatz helfen (Abb. 1).

**Abb. 2:**
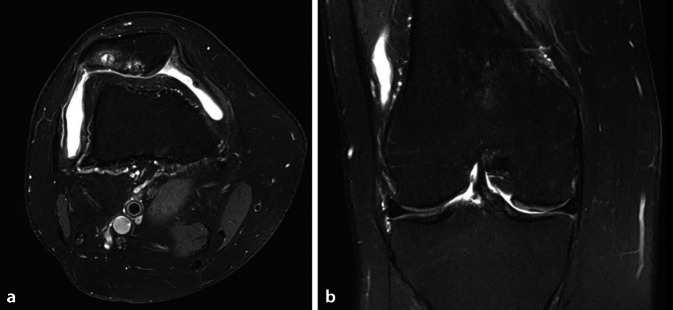
MRT (**a** axial, **b** koronar) derselben Patientin wie in Abb. 1 zum Ausschluss einer fortgeschrittenen tibiofemoralen Degeneration mit MR-tomographisch fortgeschrittener patellofemoraler Arthrose

## Patellofemorale Prothese

Die Geschichte des patellofemoralen Gelenkersatzes reicht zurück bis 1955 als erstmals eine patellofemorale Prothese (PFP) durch McKeever erfolgte. Erste Resultate eines patellofemoralen Gelenkersatzes wurden mit eher ernüchternden Resultaten im Jahr 1979 präsentiert. In den vergangenen 45 Jahren haben sich Prothesendesign und chirurgische Techniken signifikant verändert und inzwischen sind eine optimale Patientenselektion sowie eine korrekte Positionierung als wichtigste Faktoren für erfolgreiche Operationsergebnisse bei isoliertem patellofemoralem Gelenkersatz identifiziert worden [8].

Die Indikation zur PFP ist eine isolierte patellofemorale Arthrose, wenn konservative Maßnahmen für mindestens 3–6 Monate erfolgt und ausgeschöpft wurden und die Beschwerden weiter persistieren. Die Indikation kann sowohl bei primärer als auch bei sekundärer Arthrose nach Frakturen oder bei Patellamaltracking oder -instabilität gestellt werden. Insbesondere junge Patienten mit einer isolierten patellofemoralen Arthrose sind oft voroperiert und haben ein zugrundeliegendes Patellamaltracking, was die Operation und Prothesenpositionierung erschweren kann. Die Beschwerden der Patienten sollten isoliert das patellofemorale Gelenk betreffen. Schmerzen im Bereich des tibiofemoralen Gelenkspalts stellen eine relative Kontraindikation dar [8, 9].

Zur Indikationsstellung ist zusätzlich zur konventionell-radiologischen Bildgebung eine MRT nötig. Dies dient zum Ausschluss von relevanten tibiofemoralen Knorpelschäden und zur Bestätigung einer fortgeschrittenen Arthrose im patellofemoralen Gelenk. Eine Studie von Baker et al. empfiehlt die Skelettszintigraphie zur Bestätigung der isolierten PFA, da im Vergleich zur MRT eine geringere Revisionsrate festgestellt wurde, wenn präoperativ eine Skelettszintigraphie erfolgte [10].

Bezüglich eines Mindestalters für Patienten gibt es in der Literatur ähnlich zur Totalprothese keinen Konsens, wobei die PFP häufig bei jüngeren Patienten zum Einsatz kommt. Die in der Literatur beschriebene höhere Unzufriedenheit von Patienten und eine erhöhte Revisionsrate von jungen Patienten mit Knietotalprothese (KTP) ist unter anderem ein Grund, weshalb bei jüngeren Patienten die PFP der KTP vorgezogen wird. Bei jungen Patienten, die im Verlauf mit hoher Wahrscheinlichkeit eine weitere Operation benötigen, liegt ein klarer Vorteil der PFP in der sparsamen Knochenresektion am Femur, was dazu führt, dass nahezu immer im möglichen Revisionsfall einer PFP eine Standard-KTP eingesetzt werden kann. Die Konversion einer PFP auf eine Standard-KTP ist deutlich weniger invasiv und kompliziert als die Revision einer KTP. Die Schonung der tibiofemoralen Artikulation sowie der Menisken und Kreuzbänder geht mit einer natürlicheren Gelenkkinematik und besser erhaltener Propriozeption einher, wovon ebenso jüngere und aktivere Patienten potenziell profitieren [7].

Die Konversion einer PFP auf eine KTP ist weniger invasiv und kompliziert als die Revision einer KTP

Als Kontraindikationen werden in der Literatur ein Schweregrad einer tibiofemoralen Arthrose höher als Kellgren-Lawrence I, eine unkorrigierte patellofemorale Instabilität und ein relevantes tibiofemorales Malalignment (über 5° Varus oder über 8° Valgus) beschrieben [8, 9]. Zudem sind eine Patella baja (Caton-Deschamps-Index < 0,8), tibiofemorale ligamentäre Instabilität, eine systemische entzündliche Arthritis, psychogene Schmerzen, Vorliegen eines komplexen regionalen Schmerzsyndroms sowie ein Extensionsdefizit von > 10° oder eine Knieflexion von < 110° als Kontraindikationen beschrieben [8, 9]. Weitere Faktoren, die keine absolute Kontraindikation darstellen, jedoch mit einer höheren Revisionsrate einhergehen, sind ein junges Patientenalter (< 40 Jahre), Übergewicht (Body-Mass-Index > 40 kg/m^2^), das männliche Geschlecht und vorangegangene Meniskuseingriffe [8, 9].

Die Revisionsrate und funktionellen Outcomes der ersten Generation der PFP waren überwiegend optimierbar. Fortschritte in der Implantatentwicklung und der instrumentierten Operationstechnik haben die Ergebnisse heutiger Prothesen deutlich verbessert. Die Revisionsrate liegt allerdings immer noch höher als bei allen anderen Knieprothesen [11]. Nach 10 Jahren postoperativ liegt die Überlebensrate der PFP bei ungefähr 80 % [12] und die jährliche Revisionsrate bei 1,5–2 % [13]. Häufigste Ursachen für ein frühes Versagen der PFP sind persistierende Schmerzen, gefolgt vom Fortschreiten der Arthrose und Patellamaltracking. Bei der Onlay-Design-PFP wurde eine erhöhte Rate an Arthroseprogression im Vergleich zur Inlay-Design-PFP beschrieben [14]. Hauptgrund für eine späte Revision der PFP ist das Fortschreiten der Arthrose, gefolgt von der aseptischen Lockerung. Eine geringere Revisionsrate der PFP wurde beschrieben, wenn die Indikation aufgrund einer sekundären PFA im Rahmen von posttraumatischen Zuständen oder einer Trochleadysplasie gestellt wurde [15].

**Abb. 3:**
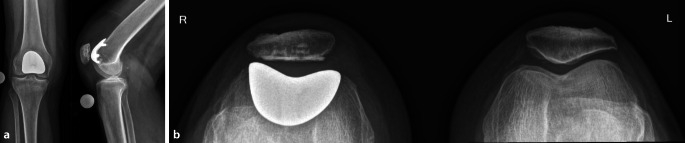
Postoperative konventionell-radiologische Bilder (**a** a. p. und lateral, **b** Patella tangential) nach Implantation einer patellofemoralen Prothese (PFP)

Die PFP ist im Vergleich zur KTP knochensparend, kann die Schmerzsituation bei isolierter PFA ausschlaggebend verbessern, hat jedoch im Vergleich zur KTP eine erhöhte Revisionsrate. Diese Eigenschaften tragen dazu bei, dass die PFP vor allem bei jüngeren Patienten (40–60 Jahre) mit fortgeschrittener PFA zum Einsatz kommt, um die Implantation einer KTP hinauszuzögern ([16]; Abb. 3).

## Knietotalprothese

Die klinischen Ergebnisse der KTP für die Behandlung der isolierten patellofemoralen Arthrose sind gut, und lange Zeit galt die KTP als der PFP überlegen, was sicherlich auch mit den schlechten Resultaten der ersten Generation der PFP zusammenhängt. Insbesondere bei älteren Patienten wurden exzellente Ergebnisse und geringe Revisionsraten mit der Implantation einer KTP zur Behandlung einer isolierten PFA erzielt [17]. Selbst bei jungen Patienten mit patellofemoraler Arthrose können gute Ergebnisse und eine hohe Patientenzufriedenheit erreicht werden, auch wenn ein Teil der Patienten nach KTP noch über persistierenden anterioren Knieschmerz klagt [16]. Ein systematisches Review von 2019 vergleicht die Ergebnisse nach KTP und PFP zur Behandlung der PFA und kommt zum Schluss, dass die PFP bei ausgewählten Patienten gleichwertige Ergebnisse erzielt wie eine KTP und zu bevorzugen wäre [18]. Dies eröffnet die Frage nach der idealen Patientenselektion.

Kamikovski et al. verglichen die Ergebnisse nach KTP und PFP an 46 Patienten unter 55 Jahren und fanden keine wesentlichen Unterschiede in den funktionellen Ergebnissen 2 Jahre postoperativ [19]. Erstaunlicherweise waren die Patienten mit KTP den PFP-Patienten ein Jahr nach Implantation überlegen und die Theorie der schnelleren Genesung nach Implantation einer Teilprothese scheint im Falle der PFP nur bedingt zuzutreffen. Die Autoren kamen zum Schluss, dass die PFP (im Gegensatz zur KTP) bevorzugt bei jüngeren, aktiven Patienten zum Einsatz kommen sollte, die von einem kinematisch normaleren Gelenk mit besser erhaltener Propriozeption profitieren. Ein Alter für die Entscheidungsfindung wurde jedoch nicht definiert [19]. Ähnlich propagieren auch Bunyoz et al. die individuelle Indikationsstellung, wobei auch sie keine klaren Kriterien zur Patientenselektion definieren [18].

Somit stellt sich die Frage, ob unter einem gewissen Alter auf die Implantation einer KTP verzichtet werden sollte und stattdessen bei einer austherapierten PFA die PFP zu bevorzugen wäre. Obwohl bei jüngeren Patienten einer niedrigere Überlebensrate und schlechtere funktionelle Ergebnisse nach KTP dokumentiert sind, ist die Revisionsrate selbst in einem Kollektiv von Patienten mit KTP unter 55 Jahren verhältnismäßig niedrig (0,5 % pro Jahr) und die Patientenzufriedenheit hoch (85,5 %) [20]. Insbesondere gilt es, den Vergleich der Revisionsrate zur PFP zu ziehen, der sich deutlich zu Ungunsten der PFP niederschlägt [21]. Es ist also abzuwägen, in welchen Fällen dieses erhöhte Revisionsrisiko der PFP in Kauf genommen werden sollte, bzw. in welchen Fällen die Implantation einer KTP zu bevorzugen ist, um den Patienten möglichst mit nur einem Eingriff ein zuverlässiges Operationsergebnis in Aussicht zu stellen. Allerdings sollte man festhalten, dass die Revisionsrate ein schlechter Parameter zur Messung einer erfolgreichen Operation darstellt, da die PFP sicherlich einfacher zu revidieren ist als eine KTP [22].

Zusätzlich sollte der körperliche Anspruch und das Alter des Patienten in Betracht gezogen werden und der PFP gegenüber der KTP insbesondere dann der Vorzug gegeben werden, wenn die Notwendigkeit einer Revision wahrscheinlich ist, zum Beispiel bei sehr aktiven Patienten unter 55 Jahren [23]. Bei fortgeschrittenem Alter und geringerem körperlichem Anspruch scheint es unserer Meinung nach nicht verhältnismäßig, auf die zuverlässigen Ergebnisse und die geringe Revisionsrate der KTP zu verzichten und die erhöhte Revisionsrate der PFP in Kauf zu nehmen, um das tibiofemorale Gelenk mit fraglichem Benefit zu schonen. Allerdings ist hier einschränkend anzumerken, dass die PFP-Operation weniger invasiv und schneller ist und damit auch bei multimorbiden betagten Patienten mit einer hohen Anzahl an Komorbiditäten sinnvoll sein kann [7].

In unseren Augen stellt deshalb die KTP in den meisten Fällen der Patienten ab 60 Jahren die Therapie der Wahl zur operativen Behandlung der isolierten PFA dar. Aufgrund der bisherigen Studienlage sollte die PFP eine Übergangslösung bleiben für junge Patienten (unter 55 Jahren) mit ausgeschöpfter konservativer Therapie bei isolierter PFA und regelrechtem, bzw. korrigiertem Patellatracking, die aufgrund einer erhöhten Revisionswahrscheinlichkeit noch nicht für eine KTP geeignet sind.

## Bikondyläre Prothese

Die Implantation einer KTP aufgrund isolierter patellofemoraler Arthrose erfolgt nach Präferenz des Operateurs mit oder ohne Retropatellarersatz. In unserem Hause ist eine KTP generell mit einem Rückflächenersatz verbunden. Insgesamt ist die Komplikationsrate für einen sekundären Retropatellarersatz nach KTP zwar gering [24], wird der Patient jedoch wegen anteriorem Knieschmerz bei patellofemoraler Arthrose operiert, wäre es nicht angemessen, nur einen Teil des patellofemoralen Gelenkes zu ersetzen. Wird auf ein Resurfacing der Patella bei patellofemoraler Arthrose dennoch verzichtet, ist meist das mögliche Komplikationsrisiko Hauptgrund für den Verzicht. Die Komplikationen eines retropatellaren Ersatzes sind zwar selten, oft aber verheerend, wobei die Patellafraktur mit/ohne Insuffizienz des Streckapparates an erster Stelle zu nennen ist. Das Risiko einer Patellafraktur nach retropatellarem Ersatz wird auf ca. 0,5–5,2 % geschätzt [25, 26]. Weitere Risiken eines retropatellären Ersatzes sind die aseptische Lockerung (0,6–4,8 %), aseptische Nekrose (< 1 %) und ein Patella-Clunk-Syndrom (6–20 %) [25, 26].

Die Patellafraktur bei einliegender KTP ist eine schwerwiegende Komplikation, kann mit dem Funktionsverlust des Streckapparates einhergehen und komplexe rekonstruktive Eingriffe nach sich ziehen. Das Risiko, eine Patellafraktur nach Resurfacing zu erleiden, steigt signifikant mit abnehmender Dicke der Patella. Bei einer Patelladicke von 20 mm kann es zu einer übermäßigen Ausdünnung der Kniescheibe unter die kritische Dicke von 12–14 mm kommen. Die Patelladicke sollte deshalb intraoperativ immer gemessen werden und im Falle einer zu dünnen Patella ist das Risiko einer Überresektion abzuwägen [27].

**Abb. 4:**
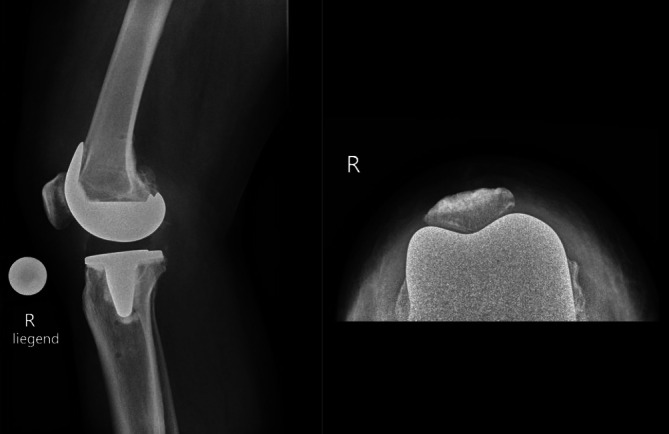
Postoperative konventionell-radiologische Bilder (**a** lateral, **b** Patella tangential) nach Implantation einer Knietotalprothese ohne retropatellären Gelenkflächenersatz bei grenzwertiger Patelladicke (12–14 mm)

Es gibt kaum Literatur zum bikondylären Ersatz bei isolierter patellofemoraler Arthrose, was die weitgehende Akzeptanz des retropatellären Resurfacings bei dieser Form der Arthrose unterstreicht. Im Jahr 2001 präsentierten Thompson et al. Ergebnisse nach KTP ohne Retropatellarersatz bei 33 Patienten mit isolierter patellofemoraler Arthrose mit einem durchschnittlichen Follow-up von 20 Monaten [28]. Anstelle eines retropatellären Ersatzes wurde durch die Autoren ein „patellar contouring“ durchgeführt, wobei die Patella mit einer Säge zurechtgetrimmt wurde, um sie wieder in eine physiologischere Form zu bringen. Es wurden allerdings keine funktionellen Scores publiziert, sondern nur ein Bericht der Schmerzfreiheit, die nur bei 63 % der Patienten erreicht wurde. Die Autoren argumentieren, dass an ihrer Institution nie ein retropatellarer Ersatz durchgeführt würde [28]. Seither haben sich die Designs der Patellaimplantate und mit ihnen auch die patellabezogenen Komplikationen signifikant verändert [13]. Zurzeit gibt es keine Daten, die den Verzicht auf einen retropatellären Ersatz bei isolierter patellofemoraler Arthrose unterstützen, sofern ein Patient eine ausreichend dicke Patella hat, die einen sicheren retropatellären Gelenkflächenersatz zulässt ([29]; Abb. 4).

## Fazit für die Praxis

- Bei isolierter patellofemoraler Arthrose kommt primär die konservative Therapie zum Einsatz. Vor Indikationsstellung zum Gelenkersatz sollte ein Patellamaltracking ausgeschlossen oder ggf. korrigiert werden.
- Eine patellofemorale Prothese kommt zur Behandlung isolierter patellofemoraler Arthrosen bei jüngeren Patienten zum Einsatz; eine klare Altersgrenze konnte in der Literatur bisher jedoch nicht definiert werden. Im Vergleich zur Knietotalprothese führt die patellofemorale Prothese häufiger zu Revisionen, und die Ergebnisse sind nicht vergleichbar mit tibiofemoralen Teilprothesen.
- Eine Knietotalprothese ist die Therapie der Wahl bei älteren Patienten (> 60 Jahre) mit isolierter patellofemoraler Arthrose und ausgeschöpfter konservativer Therapie.
- Bei stark ausgedünnter Patella sollte auf den retropatellären Ersatz verzichtet werden, um die schwerwiegende Komplikation einer Patellafraktur zu vermeiden.

## Abkürzungen

KTP: Knietotalprothese
PFA: Patellofemorale Arthrose
PFP: Patellofemorale Prothese
PRP: Plättchenreiches Plasma
